# Drug Delivery Systems from Self-Assembly of Dendron-Polymer Conjugates †

**DOI:** 10.3390/molecules23071570

**Published:** 2018-06-28

**Authors:** Burcu Sumer Bolu, Rana Sanyal, Amitav Sanyal

**Affiliations:** 1 Department of Chemistry, Bogazici University, Bebek, 34342 Istanbul, Turkey; burcusumer@gmail.com; 2 Center for Life Sciences and Technologies, Bogazici University, 34342 Istanbul, Turkey

**Keywords:** dendrimer, dendron, conjugates, nanotherapeutics, drug delivery, polymers

## Abstract

This review highlights the utilization of dendron-polymer conjugates as building blocks for the fabrication of nanosized drug delivery vehicles. The examples given provide an overview of the evolution of these delivery platforms, from simple micellar containers to smart stimuli- responsive drug delivery systems through their design at the macromolecular level. Variations in chemical composition and connectivity of the dendritic and polymeric segments provide a variety of self-assembled micellar nanostructures that embody desirable attributes of viable drug delivery systems.

## 1. Introduction

### 1.1. Nanosized Polymeric Materials as Drug Delivery Vehicles

Recent decades have witnessed an increasing trend in the application of polymeric nanomaterials for cancer therapy [1,2,3]. Widespread employment of polymer-based nanomaterials such as micellar aggregates and polymeric nanoparticles (NPs) in various application areas such as smart drug delivery systems, disease diagnosis and medical nanodevices has drawn attention to their vast potential [4,5,6]. A volume of studies in the area of polymer-based drug delivery systems has established that in most cases combining a drug to such a nanosized construct increases the efficacy of the drug. Moreover, when the drug is simply encapsulated or covalently attached as a prodrug, this happens without changing the molecular structure and the reaction of the target cells with the drug [7]. During the distribution phase, these nanoscale particles protect the drug from plasma components such as enzymes and thus preserve its stability. Since many drug molecules are quite hydrophobic, their association with hydrophilic nanocarriers increase their solubility and thus minimizes the need for additional solubilizing excipients, which can cause undesirable side effects [8,9]. Due to increased size of the carrier compared to the drug molecule, the plasma elimination half-life, tumor accumulation and renal clearance rate of the drug can be enhanced. Consequently, the therapeutic index of various chemotherapy agents which are currently in the clinic can be improved, in particular, by reducing their overall toxicity or by enhancing their efficacy [10]. Additionally, incorporation of specific targeting moieties to these nanosized aggregates facilitates delivery of the cargoes such as drugs, nucleic acids, imaging agents to specific cells or particular organelles [11,12,13]. Employing NPs to generate novel vaccines in order to improve their immunogenic response is another important area, with distinguished examples such as nicotine nanovaccines prepared from lipid-polymeric hybrid NPs displaying great potential for overcoming nicotine addiction through an innovative immunotherapeutic approach [14,15,16,17].

Although the overall picture appears quite simplistic, there are several challenges in using micellar constructs as delivery platforms [18]. When NPs are introduced or enter into biological milieus like blood stream, interstitial fluid or extracellular matrix (ECM), the coronas of particles come in close contact with biomacromolecules and adsorb them as in the case of opsonization in plasma [19]. The resulting coating around NP periphery might alter the final efficiency of drug delivery system (DDS) by changing its size, stability and surface charge [20]. As a result, numerous biological variables determining the final efficacy of the DDS such as biodistribution, toxicity, tumor extravasation and cellular internalization profiles of NPs can be distinctively different then conjectured.

Nanotherapeutic agents with an average size in the range of 10 to 200 nm can escape filtration through the kidneys, with a renal clearance cut off value around 5 nm. As a result, these NPs can stay in the blood stream circulating for prolonged periods of time. In this extra time frame, NPs have increased probability to extravasate to the tumor tissue leading to improved drug accumulation profiles [21]. The ultimate toxicity and efficacy of the delivered cargo is notably changed where the biological fate of drugs integrated to DDSs displays distinguished variances regarding pharmacokinetic (PK) properties and biodistribution of parent drugs.

Nanotherapeutics can enhance a drug’s efficacy profile during three phases; initially by interaction with the reticuloendothelial system (RES) agents while in systemic circulation; secondly during extravasation from the blood vessel towards tumor environment and finally upon uptake by target cells and release of the delivered agents. RES is a system composed of macrophages widespread in multiple organs and sites, such as lymph nodes, adipose tissue, and bone marrow, but the most essential ones are those located in the spleen and liver, since they have the greatest potential to alter the clearance profiles of NPs. The NP-corona complex formed after introduction to serum may induce recognition by macrophages and removal of these particles via phagocytic routes, as macrophages can detect the NPs coated by these serum proteins [22,23]. Thus, any aspect affecting the opsonization state of the NPs regulates the extent of interaction of the NPs with the RES components, which is the key element for gaining the preferred circulatory time and clearance rates. Surface modification of nanostructures using poly(ethylene glycol) (PEG) segments has been evaluated as an approach to address this barrier [23,24]. The PEGylation level at particle corona, the size, the composition, the surface charge and the shape of NPs are some of the features determining RES interactions. Such criterions which determine the interaction of the nanosized DDS with the biological environment and thus effecting the ultimate fate of these carriers must be kept in mind while designing a nanoparticle based carrier.

### 1.2. Dendritic Architecture-Based Drug Delivery Vehicles

The concept of three-dimensional branched polymers like dendrimers, possessing an alternative macromolecular structure compared to linear polymers was first proposed by Flory in the early 1950s [25]. However, the initial examples of this class of cascade polymeric structure was iteratively synthesized first by Vögtle and coworkers in 1978 [26], shortly followed by synthesis of lysine dendrimers in the work of Denkewalter and coworkers in 1979 [27]. Thereafter, the poly(amidoamine) dendrimers (PAMAM) were introduced through the work of Tomalia and Dewald, patented and published at 1983 and 1985, respectively [28,29]. Additionally, Newkome and coworkers in 1985 reported the monocascade sphere (arborol) type dendrimers with expendable interior cavities and modifiable surface groups indicating their potential use as micellar delivery agents [30].

Initially termed as cascade molecules or star polymers, dendrimers or dendrons are polymers with well-defined structures due to their step-by-step preparation composed of AB*_n_* type monomers (*n* usually 2 or 3) rather than the standard AB monomers which result in linear polymers. They are synthesized in an iterative manner, thereby allowing an excellent unimolecular molecular weight distribution as well as providing control over desired core and/or peripheral functionalization [31,32,33]. In addition, their branched structure offer several modifiable peripheral groups and bulky internal capacities that allow host-guest chemistry [34]. Unimolecular micelles made from dendritic domains show improved properties as low polydispersity and does not have dissociation problems due to critical micelle concentration as observed for their linear polymeric counterpart [35].

Newkome and co-workers highlighted the concept of unimolecular micelles as a stable delivery system with a hydrophobic core and hydrophilic surface [30,36]. The capacity of dendrimers to act as carriers for therapeutic cargo is enhanced by their elevated internal void volumes and it is further affected by the nature of the backbone which leads to solubilization or complex formation with its cargo through multiple intermolecular forces, such as hydrogen bonding, π-π stacking, ion-dipole interactions, or by electrostatic interactions between its surface groups and the delivered molecules [37,38]. So far, several studies have also shown that direct conjugation strategy of drugs to the dendrimer surface is feasible for increasing drug loading capacities on these type of delivery systems [39].

One of the most widely investigated dendrimer family is the PAMAM dendrimer one, which can have either amine groups or carboxylic esters as termini, depending on the growth step. Hydroxyl terminal groups can also be introduced at their periphery. The presence of reactive surface functional groups enables conjugation of drugs or targeting moieties. In addition, improved encapsulation efficiency of hydrophobic cargos is possible due to noncovalent interactions with the internal tertiary amines or through altering the core of dendrimers by incorporating hydrophobic linkers. However, the poor biodegradability profile and limited biocompatibility of PAMAM dendrimers is a major factor restraining their employment for broader biological applications. Poly (propyleneimine) (PPI)-based dendrimers are another important dendrimer family group containing amine groups at the periphery which can be altered to more biocompatible terminal groups at the dendrimer surface [40,41].

Fréchet and coworkers proposed an ether-based dendritic backbone with similar molecular structure to PEG, which provides biocompatibility to resulting dendrimers [42]. Other types of dendrimers as benzyl ether and melamine-based were also used in combination with PEG modifications to generate soluble delivery systems [43]. Another class of dendritic materials that has received a lot of attention over the past decade are the polyester dendrimers based on 2,2-bis(hydroxymethyl)propionic acid (bis-MPA) [44]. The polyester-based dendrimers composed of bis-MPA monomer units and their conjugates with PEG provide not only biocompatibility and water solubility but also biodegradability to the DDS [45]. Following the introduction of biodegradable polyester-based dendrimers, numerous monomeric building blocks such as glycerol, succinic acid, phenylalanine and lactic acid have been utilized in dendrimer backbones to achieve the generation of bio-dendrimers eligible for tissue engineering [46].

In contrast to the PAMAM dendrimers with peripheral amines leading to dose-, generation- and exposure time-dependent toxicity, dendrimers with polyester backbones display outstanding biocompatibility [47]. For this reason, it has been demonstrated that dendron-polymer conjugates containing these dendritic elements are favorable choices for many biomedical applications in recent years, including the production of novel nanotherapeutic platforms [48,49]. Furthermore, the degree of hydrophobicity can be modulated by changing the generation or peripheral functionalization of these dendrimers. In a seminal study, the importance of this particular dendritic structure in drug delivery was highlighted by Fréchet and Szoka [50]. The flexibility and ease of functionalization of polyester dendrons were validated by introducing doxorubicin (DOX) drug to their surface through acid labile hydrazone linkers. These dendrons were conjugated to a 3-arm-PEG-star core forming a high molecular weight 3-arm PEG−dendron hybrid (>20 kDa) for DOX delivery. As expected the rate of drug release was affected by pH of the environment, where acidic pH resulted in DOX release into the surrounding media while the conjugate retained its stability at neutral pH. The dendritic conjugate displayed effective cellular toxicity towards all cell lines. Importantly, in vivo experiments revealed improved plasma half-life and organ biodistribution profiles in comparison to free DOX treatments. Although, not a nanoparticulate formulation, this early example of dendron-polymer based DDS suggested the advantages of combining dendritic and polymeric building blocks.

**Scheme 1:**
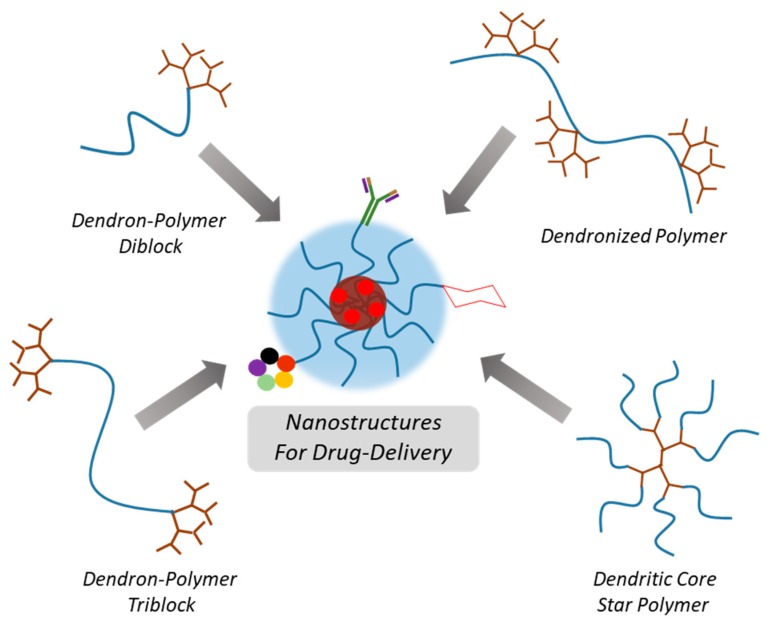
Illustration of various dendron-polymer conjugates assembling to form nano-sized drug delivery vehicles.

Understandably, the size of individual dendrimers limits the amount of drug that can be encapsulated or conjugated. Dendrimers employed as unimolecular micelles generally display hydrodynamic sizes of only a few nanometers [51]. Since plasma clearance can be regulated by increasing the size of DDS, where using constructs with high hydrodynamic volumes enables escaping from renal clearance. Thus, the approach to improve blood circulation time depends on increasing the size of the dendrimer. However, to increase size of dendritic delivery system by increasing generation is difficult since production of high generation dendrimers is rather time-consuming and it is possible to diverge from the well-defined structure at higher generations. As a solution to this problem, dendritic polymer conjugates with amphiphilic character were designed to provide larger containers like self-assembled NPs or micelles [32]. Through combination of dendritic structures with polymers, a variety of amphiphilic conjugates can be prepared (Scheme 1). Self-assembly of these polymeric conjugates in aqueous environment to nanosized aggregates provides an efficient tool to fabricate drug delivery platforms. The interior and exterior domains of these micellar aggregates can be loaded with variety of therapeutic agents and conjugated with targeting units to achieve specific delivery to the tumor site. The subsequent sections will highlight examples from literature to provide the reader with a glimpse of how dendron-polymer conjugate based polymeric materials with different architectures have been harnessed to engineer effective carriers for delivery of therapeutic agents.

## 2. Nano-Sized Aggregates from Polymer-Dendron Diblock Conjugates

Formation of micellar structures from the assembly of amphiphilic dendron polymer conjugates was reported by Fréchet and coworkers in 1992 [52]. Diblock and triblock copolymers were synthesized using a linear PEG-based hydrophilic segment and poly(aryl ether) dendron based hydrophobic segment. Mono and bifunctional PEGs were end-capped with dendrons containing a benzylic bromide unit at their focal point. Micelle formation from these constructs was probed using ^1^H-NMR in various deuterated solvents, where the two blocks possessed different solubility. Since then several dendron-polymer conjugates have been designed to serve as efficient delivery platforms. Subsequent paragraphs illustrates with a few examples to provide an overview of current state of these systems.

### 2.1. Nanoparticles Obtained with Hydrophilic Dendron and Hydrophobic Polymer Based Conjugates

In a very recent example, Wei and coworkers reported a micellar DDS system encapsulating 5-fluorouracil (5-FU) and DOX generated using an amphiphilic–dendron diblock conjugate from a generation 3.5 PAMAM dendron conjugated to a linear poly(d,l-lactide) (PDLL) block [53]. In this amphiphilic system, the dendritic moiety was hydrophilic, while the polymer segment was hydrophobic. The resulting drug loaded dendritic micelles (5-FU/Dox-DNM) displayed hydrodynamic size around 70 nm and drug loading capacity of 32% and 16% by weight for 5-FU and DOX respectively. Showing pH dependent drug release profiles as expected due to their PDLL segment, micelles were stable in cell media. MDA-MB-231 cells treated with 5-FU/Dox-DNM exhibited improved apoptotic potential compared to free drugs or single drug DNMs. Additionally, the cell populations were observed at late apoptotic quadrants indicating the synergetic potential of 5-FU/Dox-DNM formulation. When tested in MDA-MB-231 tumor xenograft models, 5-FU/Dox-DNM was able to almost inhibit tumor growth with minimal bodyweight change over the course of 14 days, even though organ accumulation of 5-FU/Dox-DNM were notable in liver, spleen and lung.

Hong and coworkers generated a micellar system based on dendron-polymer conjugates of hydrophilic PEG decorated G3 polyester dendrons conjugated to linear poly-ε-caprolactone (PCL) polymers acting as the hydrophobic block [54]. Critical micelle concentrations (CMC) of these conjugates were tuned by employing different sizes for PCL block and varying the PEG chains decorating the G3 dendrons periphery. When compared to linear micelle forming counterparts with similar hydrophilic–lipophilic balance, micelles from dendritic conjugates possessed 1–2 orders of magnitude lower CMC values. They further explored this DDS by fixing the PCL and PEG size to 3.5 kDa and 2 kDa respectively. To explore the effect of peripheral functional groups in terms of cellular interactions amine, carboxyl and acetyl terminated PEG segments were added to the study in addition to the former constructs with methoxy terminated PEGs [55]. The general characteristic were similar in terms of CMC or size, ranging between 20 to 60 nm, except that the zeta potentials of micelles with amino terminated PEGs was 23 mV, which were significantly higher in contrast to others. However, none of the constructs displayed notable difference in cell uptake of rhodamine by KB cells. The low level of internalization especially for micelles with amino terminated PEGs in contrast to the positive control PAMAM were explained as sequestration of the charges by PEG backbone, thereby inhibiting non-specific cell interactions. So decorating dendrons with PEG polymers and thereby achieving very high PEG density on micelle coronas might minimize cellular internalization regardless to surface charge of NPs. Later the authors used this DDS for topical delivery of endoxifen against breast cancer to minimize its toxic effect during oral administration [56]. Among the employed dendron-polymer conjugates, best drug loading was obtained from carboxy-terminated PEGs. Similarly carboxyl terminated vehicles displayed that these micelles enhanced endoxifen delivery through hairless mice skin samples 9 times better compared to ethanol controls, whereas liposomal endoxifen formulation achieved only 2.6 enhancement.

**Scheme 2:**
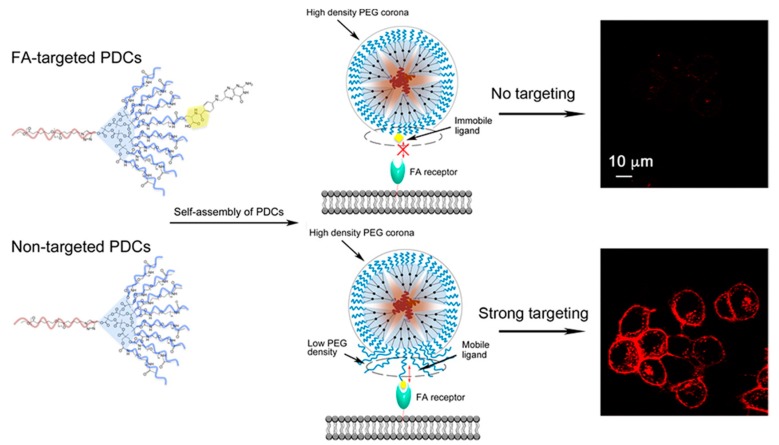
Nanoparticle formation from dendron-polymer diblocks and the effect of targeting group configuration in internalization. Adapted with permission from [57]. Copyright (2016) American Chemical Society.

A later report by the same group highlighted the importance of the disposition and the density of targeting groups on the micellar surface in affecting their interaction with cells (Scheme 2). A targeting group, folic acid (FA), was introduced to the abovementioned system and the effect of FA ligand density, cluster formation and length of PEG polymers were evaluated in terms of selectivity of cellular interactions [57]. Covalent conjugation of synthetic PEG to DDS platforms provide not only improved solubility, stability and plasma residence, but also immunogenicity of the nanostructure is reduced significantly [58]. When covered with PEG, nanoparticles are able to escape serum components and reach their destination fairly uninterrupted [59,60]. However, it was observed that the presence of high density PEG layer might hinder association with cells furthermore impair the potential of conjugated ligand by shielding them from their targeted receptors as well [61]. To overcome the “PEG dilemma” PCL-G3-PEG PEGylated dendron-based copolymer (PDC) conjugates with different PEG sizes, namely 0.6 kDa, 1 kDa, and 2 kDa and FA content were synthesized [57]. One type of each PDC and FA conjugated PDCs (PDC-FA) were then mixed at various weight ratios (0%, 5%, 10%, 25%) to obtain a family of micelles. Folate receptor overexpressing KB cells (KBFR) were treated with different rhodamine labeled micelles containing same PDC-FA weight ratio but different PEG size. The cell interactions were minimal when PEG size of PDC and PDC-FA was same, however when 0.6 kDa PEG conjugated PDC were combined with 2 kDa PEG conjugated PDC-FA the resulting DMs showed 25-fold enhanced cellular associated fluorescent values compared to non-targeted counterparts.

Another study that explored the effect of FA clusters on cell uptake utilizing a construct with polybenzyl-l-aspartate (PBLA) as hydrophobic block combined to a G4 polyester dendron with 0.6 kDa PEG chains on the periphery was reported by Hammond and coworkers [62]. They also generated a family of labeled mixed micelles with similar amount of FA ligands by combining dendritic conjugates functionalized with 0% to 100% folate groups (0%FA to 100%FA) with FA free dendritic conjugates at different weight ratios. When KBFR cells were treated with these micelles, the cell uptake increased initially by increasing cluster ratio to 20%. However, when the cluster density was further increased and weight ratio of FA containing dendritic conjugates were decreased in the final mixed micelle, cell association of these NPs decreased gradually. Three of these mixed micelles were injected to mice xenografts with two separate tumors at each flank namely the FA positive KBFR and FA negative A375 tumors. All targeted micelles showed improved accumulation in KBFR tumors relative to non-targeted controls, whereas no significant difference was reported with A375 tumors. Also, the normalized tumor fluorescence value was observed highest for mixed micelles with lowest cluster density.

### 2.2. Nanoparticles Obtained with Hydrophobic Dendron and Hydrophilic Polymer Based Conjugates

**Scheme 3:**
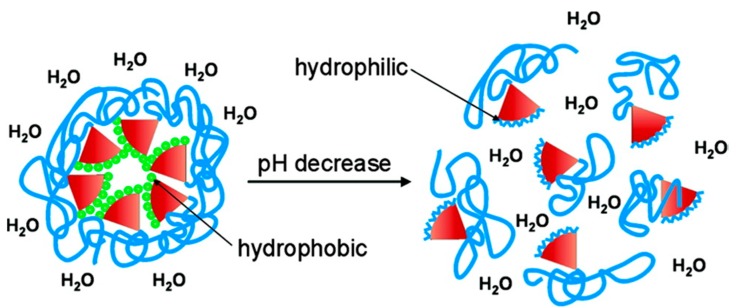
Micellar disassembly triggered by pH change. Reprinted with permission from [66]. Copyright (2004) American Chemical Society.

A different strategy that is widely followed for dendron-polymer conjugates utilizes installation of the hydrophobic dendrons at the core of the micellar structures, so that the linear hydrophilic polymer forms a moderately dense corona at the NP surface [63,64,65]. Linear-dendritic block copolymers utilizing PEG as hydrophilic block and either substituted polylysine or polyester dendrons as hydrophobic component were synthesized by Fréchet and coworkers, where the hydrophobic groups were attached to dendron peripheries via acid-labile cyclic acetal linkages [66]. These dendritic conjugates self-assembled to micelles in aqueous environment and were sensitive to pH. Micelle dissociation occurred through hydrolysis of acetals under acidic conditions, which is encountered in tumor environment or endosomal compartments (Scheme 3). Exhibiting fairly low CMC values in comparison to micelles formed from linear block copolymer, these micelles were stable at neutral pH. The dendritic biodegradable core was efficient for encapsulating various hydrophobic drugs; hence these polyester dendron based micellar systems embodied several desirable qualities of DDS platforms.

A study by Ambade and coworkers employed linear-dendritic conjugates prepared from 2 kDa PEG conjugated G2 bis-MPA dendrons decorated with alkyl chains for improved core hydrophobicity [67]. Interestingly, two stimuli responsive elements were introduced to the system, a photo-cleavable *o*-nitrobenzyl unit and an acid-labile acetal group between dendron and PEG block so that shell shedding via internal or external stimuli can be achieved to control and tune the drug delivery at the location of interest. The UV-responsive behavior of construct were demonstrated through light scattering experiments, where after 30 min UV irradiation a shift in hydrodynamic size from 90 to 30 nm was observed. Incubation of these NPs in acidic buffer for 7 days resulted in minor increase in degradation at pH 5.0 showing slower cleavage potential of acetal linkages relative to *o*-nitrobenzyl groups. The cellular uptake of DOX was also studied and a 30 min UV irradiation led to an increase of DOX incorporation by 40%. Another light responsive system was reported by Dong and coworkers who reported fabrication of micelles from linear-dendritic diblock constructs composed of generation 3 PAMAM dendron decorated with hydrophobic dye diazonaphthoquinone (DNQ) and linear PEG [68]. Exposure of micelles to UV or near infra-red (NIR) irradiation disrupted the micelles to release the encapsulated drug, doxorubicin, through transformation of the hydrophobic DNQ fragment to a hydrophilic 3-indenecarboxylic acid unit via the Wolff rearrangement. The NIR-triggered cytotoxicity of the drug loaded micelles was also demonstrated through in vitro studies with HeLa cells.

Malkoch and coworkers developed a micellar DDS using similar amphiphilic linear-dendritic polymeric conjugates utilizing rhodamine bound 10 kDa PEG tail conjugated to a G4 polyester dendron decorated with 16 cholesterol groups. Cholesterol, a naturally occurring highly hydrophobic bulky lipid was chosen to improve the drug loading capacity as well as micelle core stability [69]. The linear-dendritic conjugates formed micellar aggregates (NC20) with hydrodynamic size of 172 nm and DOX loading content of 18.8 ± 1.3 by weight. Decrease of mitochondrial function indicating the loss of cell viability was explored in drug resistant resi-MCF-7 cell line. DOX-NC20 micelles showed significant reduction in mitochondrial function in comparison to DOX only controls. An additional decrease was also achieved by co-delivery of triptolide (TPL) drug with DOX in TPL-DOX-NC20 micelles.

Dendrons other than those based on bis-MPA have also been employed as a biodegradable block. In a recent study Wei and coworkers used a linear-dendritic conjugate that was synthesized by combining methoxy-terminated PEG (mPEG) and glycolic acid oligomer-based, 4-benzyloxy-4-oxobutanate terminated G3 polyester dendrons [70]. DOX encapsulated micelles were prepared with drug content of 21.2% and size around 150 nm. These micelles displayed pH dependent DOX release profiles due to their acid sensitive polyester backbones. DOX release increased to 39% when incubated at pH 5.0 buffer, as compared to 20% release upon incubation at pH 7.4 for 96 h.

**Scheme 4:**
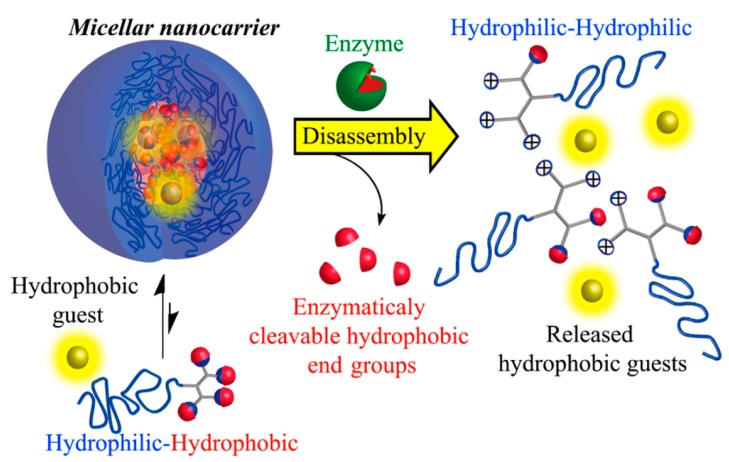
Micellar disassembly and drug release via PGA enzyme cleavage. Reprinted with permission from [71]. Copyright (2014) American Chemical Society.

In a very elegant approach, an enzyme cleavable linear dendritic polymer conjugate system was reported by Amir and coworkers [71]. Different size PEG blocks (2, 5 or 10 kDa) were conjugated to a generation 2 dendron which was decorated with four phenyl acetamide molecules at the periphery acting as ligands to penicillin G amidase (PGA) enzyme (Scheme 4). The hydrodynamic size of assembled micellar NPs were determined before and after PGA incubation where dissociation of 2 and 5 kDa PEG micelles was completed after 8 h, whereas it took only 4 h for 10 kDa PEG micelles. Moreover the dissociation rates by fluorescence assays indicated faster enzyme triggered disassembly for micelles with longer PEG chain at the corona explained by a hypothesized equilibrium between “monomeric” and micellar polymeric states. In a subsequent study, the authors reported a micellar system with lower CMC and enhanced stability towards enzymatic degradation by introducing a thiol group on the dendritic fraction. The reversible dimerization of the thiol group to form disulfides imparts the added stability to these self-assembled nanostructures [72].

Lam, Luo and coworkers employed PEG (5 kDa) conjugated to a third generation of dendritic polylysine block which was further modified with cholic acid, to generate an amphiphilic PEG5k-CA8 telodendrimer [73]. This linear-dendritic block copolymer achieved high paclitaxel (PTX) encapsulation capacity up to 35% by weight and displayed stability over six months through addition of cholic acid that is a natural surfactant. Biodistribution studies in female athymic nude mice with SKOV3-luc cells subcutaneous xenografts indicated an almost 2.5 fold increase at 12 h in accumulation of DiD (1,1′-dioctadecyl-3,3,3′,3′-tetramethylindodicarbocyanine 4-chlorobenzene-sulfonate) dye loaded DiD-PTX-NPs in tumor environment in comparison to free dye treatment and this difference was maintained at 72 h. DiD fluorescence levels at multiple tissues were evaluated ex vivo highlighting again improved tumor accumulation but also with notable lung and liver accumulation as well. However, PTX-PEG5k-CA8 NPs displayed improved therapeutic activity in both subcutaneous and orthotopic ovarian cancer models compared to Taxol^®^ as well Abraxane^®^ with evident decrease in tumor volume or increase in median survival days.

**Scheme 5:**
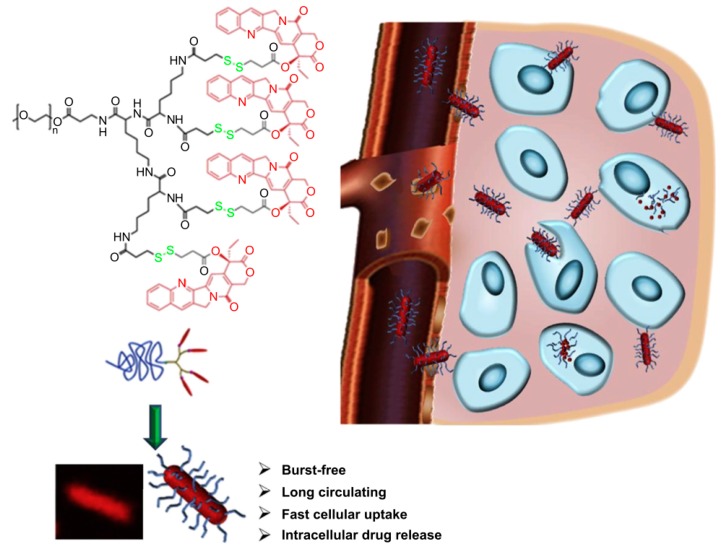
Illustration of internalization of DOX loaded sized PEG_45_-OctaCPT nanorods. Reprinted with permission from [74]. Copyright (2013) Elsevier.

In an interesting study, Shen and coworkers demonstrated nanorod formation of linear dendritic conjugates composed of a 2 kDa mPEG segment attached to generation 2–3 polylysine dendrons bearing camptothecin (CPT) drugs on their periphery attached through disulfide linkers (Scheme 5) [74]. Depending on dendron, CPT incorporation as high as 38.9% was possible. CMC values also showed generation dependence and they were as low as 0.025 mg/mL for micelles prepared from G3 dendron containing conjugates (PEG_45_-OctaCPT). DOX was also loaded to micelle to evaluate internalization by MCF7/ADR cell and a dendron generation dependent fluorescence signal increase was noted for PEG_45_-xCPT/DOX micelles. Biodistribution and pharmacokinetic (PK) properties of NPs were also tested. PEG_45_-TetraCPT nanorods with G2 dendrons showed highest plasma clearance half-life of 5.82 h and all micelles showed spleen accumulation after 24 h. The ex vivo imaging of dissected tumors indicated poor tumor accumulation for micron-sized PEG_45_-OctaCPT rods, whereas highest accumulation was reported for PEG_45_-TetraCPT with medium lengths (<500 nm).

**Scheme 6:**
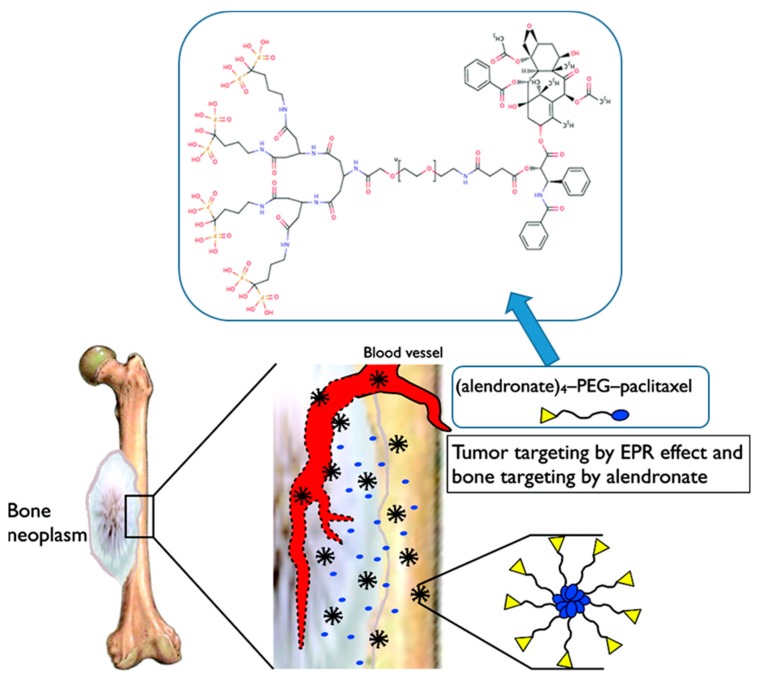
Illustration of bone tumor targeted micellar constructs from PTX-PEG-ALN. Adapted with permission from [75]. Copyright (2011) American Chemical Society.

Pasut and coworkers reported β-glutamic acid dendrons to introduce multiple bone targeting groups. A linear dendritic construct was prepared by conjugating a 5 kDa PEG polymer with an amino-bisphosphonate alendronate (ALN) modified generation 2 β-glutamic acid dendron, where PTX was attached to the other end of PEG through an ester group [75]. The ALN groups were utilized both to target the micellar assemblies of PTX-PEG-ALN conjugate to bone through high affinity of ALN towards bone-mineral hydroxyapatite (HA), as well as to exert apoptotic and anti-angiogenic effect towards bone metastases (Scheme 6) [75]. On Matrigel environment both the non-targeted PTX-PEG and targeted PTX-PEG-ALN micelles were able to inhibit tube formation of HUVECs up to 50%. Similar reduction in microvessel density for both micellar formulations were reported from immunohistochemical analysis of 4T1 tumors in tibia. However, an enhanced apoptotic effect was reported of PTX-PEG-ALN micelles by the significant increase in counts of apoptotic circulating endothelial cells detected in blood indicating the selective targeting of these NPs against metastatic cells. Targeted micelles also displayed improved accumulation after 8 h of injections in MDA-MB-231 mammary tumors in mice tibia, and longest plasma elimination half-lives, resulting in 50% tumor growth inhibition compared to saline controls [76].

While a majority of studies reported to date have utilized PEG-based polymers as the hydrophilic segment, a few studies have also focused on utilization of other hydrophilic polymers. Recently, Bi and coworkers reported thermoresponsive micellar aggregates prepared from diblock constructs composed of poly(benzyl ether) dendrons and linear poly(*N*-vinylcaprolactam) [77]. Dendritic poly(benzyl ether) blocks containing a benzyl chloride group at their focal point were used as initiators for atom transfer radical polymerization of *N*-vinylcaprolactam. The self-assembled structures in aqueous media varied from irregular spherical micelles, vesicles to rod-like large compound vesicles depending on the generation of the dendritic component. These amphiphilic copolymers could undergo thermally-induced phase transition in aqueous media. These copolymers were found to be nontoxic toward mouse L929 fibroblast cells, thus suggesting their biocompatible nature. As an alternative to PEG as a hydrophilic component, Hoogenboom and coworkers reported the synthesis of dendron-polymer diblock conjugates obtained through conjugation of hydrophilic poly(2-ethyl-2-oxazoline) polymer to acetal protected hydrophobic polyester dendrons [78]. Degradation of micelles at acidic pH was confirmed through light scattering experiments.

### 2.3. Nanoparticles Obtained with Hydrophilic Dendron and Hydrophilic Polymer Based Conjugates

Gene therapy is another area for employment of dendrimers since they can provide well-defined, compact structures decorated with multiple terminal groups where surface functionalization with relevant moieties can be achieved easily [79]. As an alternative to viral vector systems which suffer from problems such as high toxicity and immunogenicity [80], cationic polymers especially dendrimers and dendritic conjugates can from stable complexes with nucleic acids such as plasmid DNA or siRNA via multivalent electrostatic interactions. The electrostatic complex formation eliminates the need for amphiphilic copolymers [81]. Since nucleic acids carry negative charge that challenges cellular internalization, and also can be cleared from blood stream rapidly by serum nucleases [80], it is crucial to combine them into dendriplexes to achieve safe and efficient transfection to targeted cells [82,83].

An example of polymer-dendron diblock conjugates for genetic material reported by Hammond, Langer and coworkers focused on generation of a PAMAM-PEG dendritic copolymer family for targeted delivery of plasmid DNA (pDNA). Different generations of PAMAM dendron were screened and it was observed that polyplex formation was favored by increasing generation due to higher charge density [84]. Furthermore, by addition of mannose or galactose targeting groups through the PEG segment increased transfection efficiency. Improved transfection of HepG2 hepatocytes via galactose targeted PAMAM polyplexes compared to commercially available PEI counterparts was observed. 

**Scheme 7:**
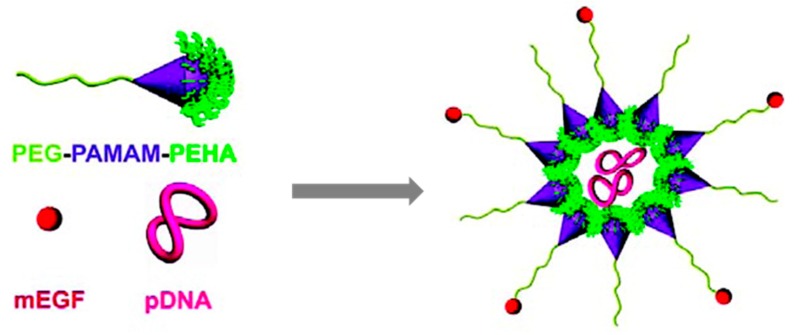
Delivery of pDNA using a non-amphiphilic dendron-polymer conjugate. Reprinted with permission from [85]. Copyright (2011) American Chemical Society.

These constructs are very versatile since it is possible to modify the dendron periphery as well as the linear polymeric part to add desirable properties to these DDS. Yu and coworkers reported a mouse endothelial growth factor (mEGF) targeted dendron-polymer conjugate for delivery of pDNA (Scheme 7) [85]. Generation 3.5 PAMAM dendrons were modified with different oligoamines to improve their transfection efficiency, where best results were provided by the pentaethylenehexamine (PEHA) substituent. The PAMAM-PEHA conjugates were further modified with EGF-PEG block to benefit from the anti-biofouling effect of PEG and active targeting of EGF receptor overexpressing tumor cells. The final EGF ligand conjugated polyplexes, bearing luciferase pDNA in their core were able to selectively improve gene delivery by 10-fold on EGFR overexpressing HuH-7 cells in comparison to their non-targeted counterparts.

It must be noted that while oligonucleotide based materials have been used as a cargo, dendrimeric constructs composed of oligonucleotides have been synthesized. For example, Tomalia and coworkers used complementary DNA strands at focal points of dendrons to assemble dendrimers through self-assembly [86]. Likewise, other dendrimer structures that are purely composed using oligonucleotides have been synthesized by various groups [87,88].

## 3. Nano-Sized Aggregates from Polymer-Dendron Triblock Conjugates

As mentioned earlier, formation of micellar structures from diblock and triblock copolymers was reported by Fréchet and coworkers in 1992 [52]. A more detailed study of amphiphilic dendron-polymer-dendron triblock conjugates assembly was later reported by Gitsov and coworkers [89]. The construct was composed of linear PEG chain flanked by hydrophobic dendritic poly(benzyl ester)s and was synthesized using a divergent growth strategy. It was observed that while the conjugates with second generation dendrons with hydrophobic groups at their exterior self-assembled in water at low concentrations (10^−6^ mol/L), their counterparts where the peripheral groups were removed to expose hydroxyl groups were soluble under similar conditions. The authors postulated the potential of these self-assembled aggregates for possible applications in drug delivery.

**Scheme 8:**
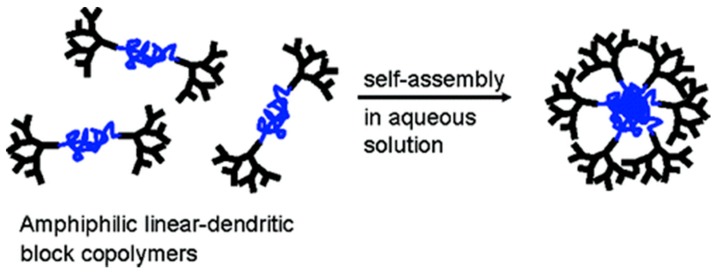
Self-assembly of ABA-type dendron-polymer-dendron conjugate with hydrophilic dendrons and hydrophobic middle segment. Reprinted with permission from [90]. Copyright (2006) American Chemical Society.

This study was followed by a report by Nguyen and Hammond who reported the assembly of a ABA-type dendron-polymer-dendron conjugate composed of PAMAM dendrons as hydrophilic components and poly(propylene oxide) as the hydrophobic middle block [90]. Notably, such constructs provide structures where the dendritic wedges are on the outside of the nanostructure as opposed to being buried inside as in abovementioned example (Scheme 8). PAMAM dendrons were divergently synthesized from an amine group containing telechelic poly(propylene oxide) polymer. Assembly in water resulted in formation of nanoparticles in the size range of 9–18 nm. To demonstrate its utility as a viable drug delivery system, a hydrophobic drug, triclosan, was encapsulated with loading efficiencies of 79–86% *w*/*w*.

**Scheme 9:**
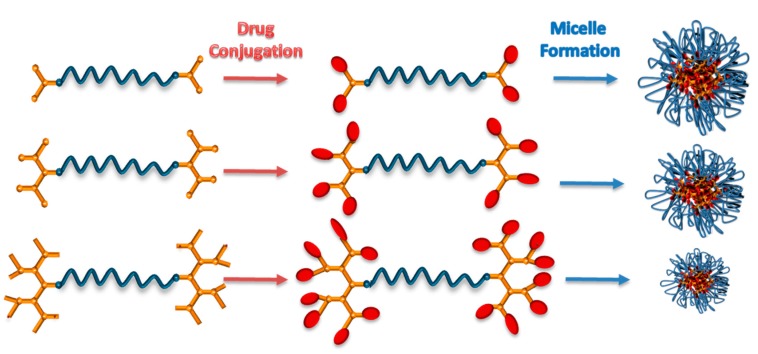
Illustration of combretastatin-A4 conjugation to ABA type dendron-polymer-dendron conjugates and micelle formation.

Sanyal and coworkers used an ABA type dendron-polymer-dendron conjugate with 10 kDa middle PEG segment connecting two identical polyester dendrons with generation 1–3. The dendron surfaces were further modified with combretastatin-A4 anti-angiogenic drug (Scheme 9) [91]. Size and CMC values of prepared Comb-Gx-PEG and Gx-PEG micelles displayed dendron generation and drug content related trends. Even though combretastatin-A4 release occurred faster at acidic pH as expected, it took approximately 4 days to achieve high cumulative release results even at acidic environment, which indicates their potential as a slow drug releasing system. The Comb-G3-PEG micelles were further exhibited dose dependent anti-angiogenic effect in tube formation assay on HUVECs.

**Scheme 10:**
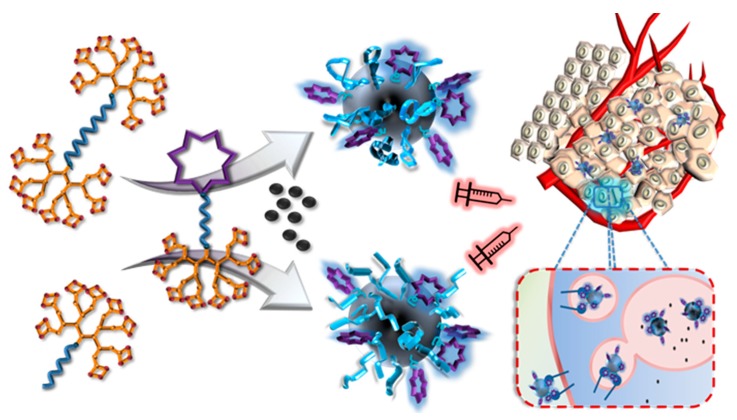
Illustration of drug loaded, RGD targeted micellar NPs via “Mix-and-Match” approach employing AB and ABA type dendron−polymer constructs. Reprinted with permission from [92]. Copyright (2017) American Chemical Society.

Nanostructured delivery agents can also be fabricated using a combination of diblock and triblock dendron-polymer conjugates. In a recent study, Sanyal and coworkers reported a modular micellar system for delivery of docetaxel. An AB type diblock dendron polymer conjugate was used to introduce targeting RGD (arginine-glycine-aspartic acid) peptides onto NP surface (Scheme 10) [92]. The peptide targeting group containing diblock copolymer was composed of a linear PEG polymer appended with an acetal protected hydrophobic polyester dendron at one end and the cyclic RGD peptide targeting unit at the other end. Either an ABA type triblock conjugate composed of two acetal protected G4 polyester dendrons with a 6 kDa PEG middle segment or an AB type copolymer composed of a G4 polyester dendron conjugated to a 2 kDa mPEG polymer was mixed with the diblock polymer-dendron conjugate modified with the RGD peptide. Both AB and ABA PEG-based micelles possessed similar CMC values and drug loading contents with hydrodynamic sizes in the range of 170–230 nm. While both AB and ABA micelles were stable even after more than 1000-fold dilutions beyond their CMCs, only the AB micelles could retain their size in the presence of 10% FBS, whereas aggregate formation was observed for the ABA system. The cell internalization studies indicated that AB micelles showed higher association with MDA-MB-231 cells relative to the ABA ones, while presence of RGD increased cellular internalization in both cases.

Although a majority of studies to date have focused on the delivery of anti-cancer drugs, these dendritic conjugates can also be utilized as delivery platforms for drugs against different diseases such as malaria. To overcome the high drug resistance of malaria parasites, provide improved cellular delivery of antimalarial drugs and achieve sustained drug release micellar assemblies were prepared using different Janus-like dendrons or ABA type dendritic-linear-dendritic conjugates [93]. Enhanced cellular association and decreased IC_50_ values for primaquine and chloroquine were reported for assemblies formed using dendritic-linear-dendritic conjugates. Additionally, improved plasma circulation times and survival times were noted relative to free drugs in malaria infected mice.

## 4. Nano-Sized Aggregates from Star Type Architectures

The star shaped structure of a dendrimer allows them to act as drug carriers though two different modes. These structures can either encapsulate drug molecules in their internal voids through non-covalent interactions, or the drug can be covalently conjugated onto their periphery. Dendrimers like PAMAM and PEI have been utilized to encapsulate drug molecules through stabilizing electrostatic or hydrogen bonding interactions [94,95]. Another example includes, biodegradable dendrimers based on glycerol and succinic anhydride reported by Grinstaff and coworkers which could encapsulate various camptothecin derivatives, with exhibited superior in vitro performance than free drugs [96]. Attachment of drugs through non-covalent and covalent modes have both pros and cons and such comparisons have been recently reported in terms of drug release profiles, systemic toxicity and anti-tumor efficacy [97,98]. The limitation in drug loading, as well as facile release from dendrimers due to their small size promoted modification of dendrimer peripheries with polymer chains. Several drug delivery vehicles based on PAMAM dendrimers modified with PEG chains and other hydrophilic polymers have been reported to date [99,100,101]. Additionally, attachment of PEG also reduces the toxicity of PAMAM dendrimers containing amine groups at the periphery [102,103]. Furthermore, improving their in vivo performance such as prolonged circulation time and increased accumulation at tumor site is also possible, e.g., upon modification with PEG chains bearing targeting groups such as folic acid [104]. It must be noted though that such dendrimer-polymer constructs generally act as unimolecular micellar containers. Since the focus of this review is self-assembled nanostructures based on conjugates of dendron/dendrimer with polymers, such unimicellar constructs are not included here. Micellar constructs from star shaped amphiphilic constructs with dendrimers as core unit are rare. Fang and coworkers reported star shaped polymers composed of a G2 PAMAM core, from which a hydrophobic poly(ε-caprolactone) was grafted from using ring-opening polymerization, followed by conjugation to linear PEG chains as outer blocks [105]. These 16-arm PAMAM cored star polymers produced a mixture of unimolecular micelles and micellar aggregates as determined using dynamic light scattering experiments. Anti-cancer drug indomethacin, doxorubicin and etoposide loaded micelles were prepared using either dialysis or oil/water emulsion method. The authors reported a subsequent study where they used a 32-arm PAMAM dendrimer as a core to synthesize PAMAM-PCL-PEG and PAMAM-PLA-PEG star polymers containing the PCL and poly(l-lactide) (PLA) based middle blocks, respectively. These polymers also yielded a mixture of unimolecular micelles and micellar aggregates in aqueous environment, which could be loaded with an anti-cancer drug, etoposide, with higher loading in the PCL-containing construct. Interestingly, the 32-arm PAMAM-PCL-PEG constructs exhibited lower drug loading for etoposide than the 16-arm counterpart, which authors suggest could be a result of higher PCL chain density in the star polymers obtained with the higher generation dendrimer [106].

**Scheme 11:**
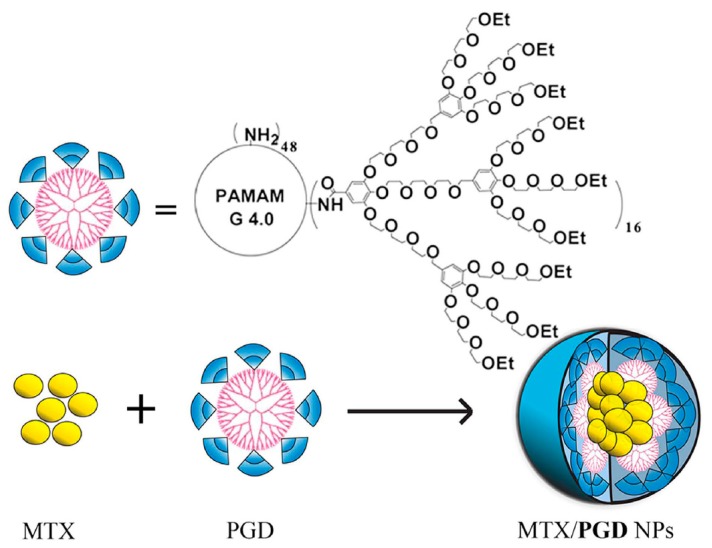
Structure of unimeric PGD and MTX encapsulating MTX/PGD nanoparticles. Reprinted with permission from [109]. Copyright (2016) Springer Nature.

Another interesting architecture that has been recently reported is not strictly a star like linear polymer-dendrimer construct but a dendron appended dendrimer. This example cleverly illustrates that it is possible to utilize two types of dendritic fragments in the same delivery platform to benefit from characteristics of both types. Wang and coworkers developed a methotrexate (MTX) encapsulating PAMAM and oligoethylene glycols dendron (PGD) based co-dendrimer through conjugation of oligoethylene glycols (OEG) dendrons onto a G4 PAMAM dendrimer surface (Scheme 11) [107]. The PAMAM core in the dendronized dendrimer PGD provides as a reservoir for MTX. Moreover, the OEG dendrons act as solubilization agents, as well as they can diminish the cytotoxic potential of PAMAM dendrimers. The MTX release displayed dependence on the thermosensitive characteristics i.e., temperature dependence of solubility of the OEG dendrons. Also depending of concentrations, the PGD particles displayed a hydrodynamic size of 8 nm for unimeric form and around 110 nm for aggregates. They also reported that the number of conjugated OEG dendrons or decoration degree affect the drug loading capacity and the interaction of PGD NPs with cells. Among the various co-dendrimers, best results were obtained for those with decoration number 16 and 32 [108]. The authors further optimized the drug loading content up to 85.2% via employing an anti-solvent precipitation method augmented by ultra-sonication [109]. The MTX/PGD NPs displayed improved cytotoxic profiles both in MCF-7 and 4T1 cells in comparison to MTX only. Similarly, internalization of cy5.5 loaded PGD NPs showed significant cell uptake. In vivo tumor inhibition studies using BALB/c mice 4T1 breast tumor xenografts showed significant tumor growth limitation even at 2 mg/kg MTX/PGD NPs. On the other hand, the control samples displayed 23-fold volume increase whereas tumor volume increase with 8 mg/kg MTX/PGD NPs treated mice was 9.6-fold in 10 days. For evaluating biodistribution of NPs ex vivo fluorescent imaging studies indicated significant increase in tumor accumulation of NPs however similar increase in NP accumulation was also observed with organs of RES as liver, spleen, and kidneys.

## 5. Nano-Sized Aggregates from Miscellaneous Dendron-Polymer Architectures

Dendronized polymers are another emerging class of macromolecular architecture that have been frequently utilized in recent years to obtain nanomaterials for drug delivery. These constructs comprise of dendrons conjugated as side chain residues along the backbone of a polymer. In a simple approach, amphiphilic constructs are obtained when hydrophilic synthetic or natural polymers are appended with dendrons that are either inherently hydrophobic or become so upon attachment of hydrophobic drug molecules.

**Scheme 12:**
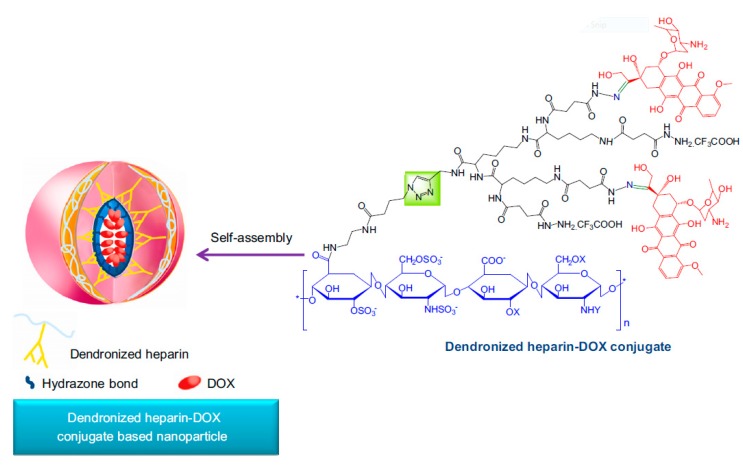
Illustration of self-assembly of DOX conjugated dendronized heparin NPs. Reprinted with permission from [110]. Copyright (2013) Elsevier.

In a recent example, Gu and coworkers generated dendronized polymers composed of heparin backbone decorated with G2 lysine-based dendrons bearing DOX molecules (Scheme 12) [110]. A Huisgen type ‘click’ reaction between an azide-containing heparin and lysine-based dendrons bearing an alkyne at their focal points catalyzed by a Cu(I) catalyst yielded the dendronized polymers. Interestingly, even prior to the attachment of drug, the parent dendron-heparin conjugates self-assembled into NPs with hydrodynamic size of 250 nm, presumably due to hydrogen bonding interactions between the charged amine groups on the dendrons with the sulfo and carboxyl groups on the biopolymer. Upon conjugation of the drug, the size of NPs decreased to 90 nm, which was attributed to introduction of additional interactions such as pi-pi stacking etc. from the drug. As expected, drug release in a pH responsive manner was observed, since acid-sensitive hydrazone linkages were used for conjugating DOX to dendrons periphery. High levels of anti-angiogenic and anti-tumor activity for the drug loaded NPs compared to free drug was demonstrated through in vivo experiments.

The same group reported another dendronized copolymer composed of a 20 kDa dextran decorated with fluorinated G2 lysine dendrons as side chains conjugated to polymer backbone via acid-labile hydrazone linkers [111]. Dextran was chosen for providing improved biodistribution for bioconjugates, and trifluoromethyl groups were introduced on the dendrons in order to increase hydrophobicity of the construct to enable efficient encapsulation of hydrophobic drug molecules. The drug loading content of DOX was 20.4 ± 3.1 for this system and the drug was released in a pH sensitive manner.

In an alternative assembly strategy, Gu et al. used a G3 lysine dendron functionalized with DOX via hydrazone linkages appended with two mPEG tails at the dendron focal group [112]. The dendron-PEG conjugates with 14% drug content assemble into NPs with hydrodynamic size of ~220 nm and possess a neutral surface charge. In contrast to the negligible drug release at physiological conditions, incubation at pH 5.0 led to an initial burst release and more sustained type release profile reaching up to 80% in 54 h. Antitumor activity of DOX NPs was studied through treatment of mice with 4T1 breast tumors every four days for 13 days. Significant inhibition of tumor growth, as well as almost no decrease in body weight of treated mice were reported for DOX NPs relative to saline control and free DOX treatments.

**Scheme 13:**
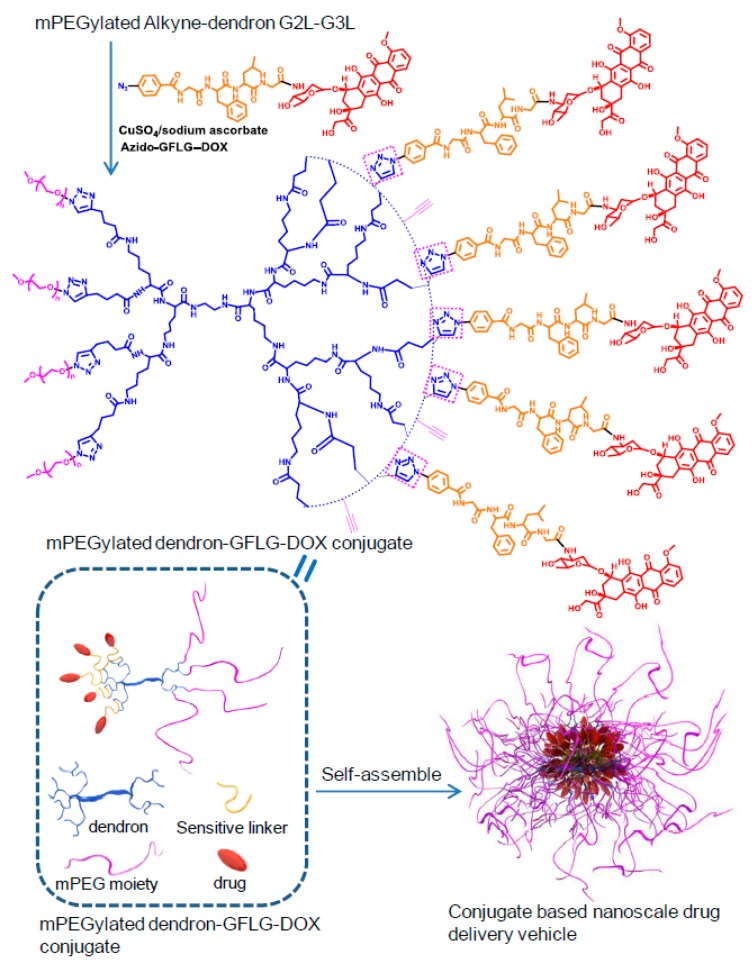
Illustration of self-assembly of PEGylated GFLG-DOX conjugated lysine dendrons. Reprinted with permission from [113]. Copyright (2014) Elsevier.

In a subsequent study, the authors further modified this NP system by using a G2 lysine dendron and thickening their PEG corona by using four 2 kDa PEG tails [113]. Additionally, instead of acid labile hydrazone linkers for dendron functionalization, an enzyme responsive GFLG peptide based linker was employed (Scheme 13). This allows triggering of drug release after cellular internalization since the GFLG peptide sequence is cleaved by the enzyme cathepsin B, a lysosomal cysteine protease that is upregulated in various tumors [114]. DOX-conjugated GFLG responsive NPs with 9.6% drug loading content were shown to release their cargo strictly in the presence of sequence specific proteases. The NPs displayed significant tumor growth inhibition relative to free DOX and saline controls. Moreover, the anti-angiogenic and especially anti-apoptotic effect of these drug loaded NPs was confirmed via immune-histochemical staining assays.

A conceptually different type of dendronized polymers was reported by Guan and coworkers [115]. They employed polymeric constructs containing peptide-based dendrons as siRNA delivery agents. In an interesting approach, they generated a linear peptide backbone consisting of dicysteine and l-lysine units thereby introducing an oxidation responsive disulfide linkage along the polymeric back bone. Thereafter, the backbone was further modified with G1 or G2 poly(l-lysine) dendrons with peripheral modifications with different hydrophobic or hydrophilic amino acids, thereby creating a combinatorial library of dendronized peptide polymers for siRNA delivery to NIH 3T3 cells.

The siRNA complexation with various dendronized polymers resulted in spherical NPs with diameters below 100 nm, which disintegrated upon treatment with glutathione (GSH) and released the siRNA. The in vitro transfection efficiency of NIH 3T3 cells with an anti-GFP siRNA demonstrated that polyplexes formed with the G2 construct containing 75 mol% histidine and 25 mol% tryptophan residue at dendron periphery at 80 primary amine/siRNA phosphate (N/P) ratio were able to provide improved GFP down regulation especially at lower serum conditions in comparison the lipofectamine. The effect of different amino acids at dendron periphery on the complex formation and transfection efficiency was evaluated.

**Scheme 14:**
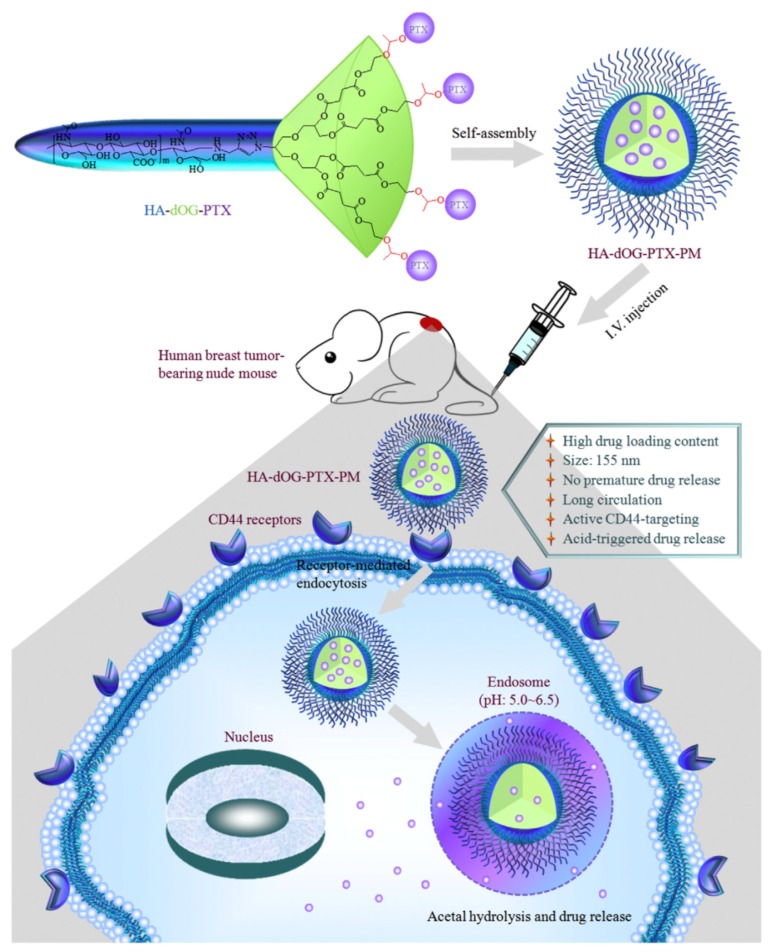
Micellar constructs based on polyglycerol-hyaluronic acid conjugate. Reprinted with permission from [116]. Copyright (2016) Elsevier.

Dendritic polyglycerol (dOG) unit appended polymers are appealing building blocks with polyether internal cavities, biocompatibility similar to PEG and multiple hydroxyl groups enabling surface functionalization. Haag and coworkers conjugated polyglycerol dendrons modified with paclitaxel (PTX) through acetal linkages to hyaluronic acid (HA) to prepare HA-dOG-PTX-PM micelles. The HA shell serves as a protective natural polysaccharide layer, as well as enables active targeting of CD44 positive cancer cells (Scheme 14) [116]. The micellar assemblies dissociated under acidic environment, as suggested from DLS analysis, and triggered the release of PTX. Internalization of DOX loaded HA-dOG-PTX-PM by CD44 positive MCF7 cells were higher than free DOX, and a notable decrease in DOX internalization was observed by addition of excess HA as a competitor. Similar improvements in PK and biodistribution of micellar PTX was also reported; for instance, plasma elimination half-lives of micellar PTX and Taxol was 4.32 and 0.23 h, respectively. The therapeutic effect of micellar constructs were studied on nude mice bearing MCF-7 tumor xenografts. Excellent tumor progression inhibition with no eminent body weight loss was noted, thus highlighting the therapeutic potential of this PTX delivery system against CD44 positive breast tumors.

**Scheme 15:**
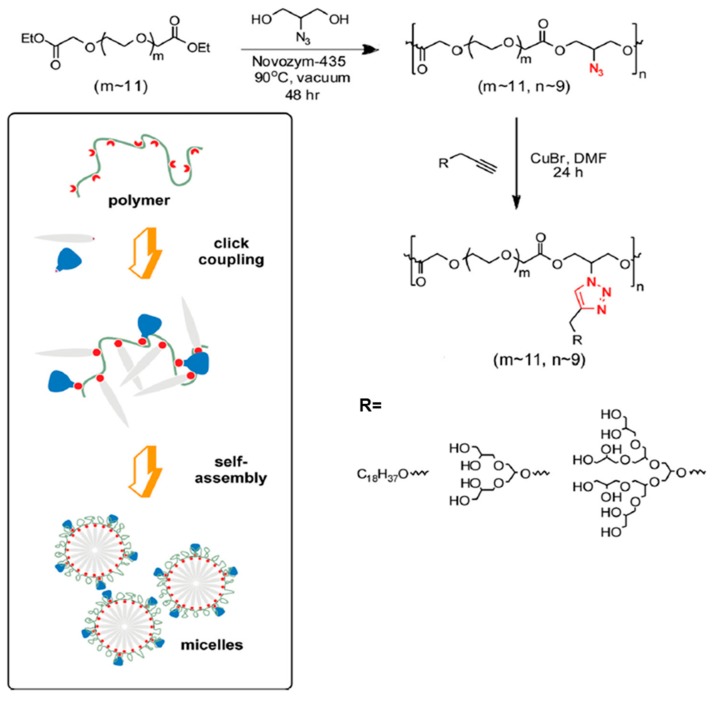
Synthesis of dendronized polymers and their self-assembly into NPs. Reprinted with permission from [117]. Copyright (2013) WILEY-VCH Verlag GmbH & Co. KGaA, Weinheim.

The dendritic polyglycerol unit has also been used as side chain appendages in dendronized polymers. A hydrophobic polymer bearing glycerol dendrons as side chains self-assemble to generate micellar NPs (Scheme 15). Haag and coworkers used click chemistry to conjugate dendritic polyglycerols dendrons modified with octadecyl chains to an enzymatically synthesized condensation polymer from azido-glycerol and PEG600 diethyl ester [117]. Obtained NPs displayed low hydrodynamic sizes ranging between 14–20 nm, with polydispersity index (PDI) values between 0.427–0.603. Their potential as drug delivery agents were evaluated by pyrene encapsulation experiments. In a following study, similar constructs with PEG1000 diethyl ester and an additional hyperbranched polyglycerol dendron was used and a set of self-assembling dendronized polymers were prepared [118]. QGP-1 cells were treated with Nile red encapsulated dendronized polymers displayed enhanced dye incorporation than free Nile red or Nile red loaded linear polymer-dendron diblock conjugate counterparts. Furthermore these micellar NPs demonstrated minimal cytotoxicity at concentrations as high as 500 μg/mL after 72 h. A system using fluorinated version of the hydrocarbon chain was later used as a curcumin delivery system [119].

A recent study from Imae and Chung and coworkers demonstrated that apart from using homopolymers components, diblock polymers where one of the block is dendronized can be used as building blocks for NPs effective for drug delivery. A linear polyelectrolyte block conjugated to a hydrophobic block with pendant dendrons assembled into micelles [120]. DOX loading capacities as high as 95% was reported. Interestingly, these micelles showed improved cytotoxicity with lower IC_50_ of 0.12 μM against 4T1 cells, compared to free DOX. Usually, in general, the free DOX is observed to possess more cytotoxicity in in vitro studies. Drug loaded micelles demonstrated 1.5 fold improved tumor growth inhibition relative to free DOX in BALB/c mice bearing 4T1 mammary tumors [121].

The brief survey of the abovementioned diverse polymeric structures that can be obtained through combination and dendrons and polymers, and are different than the classic AB and ABA type block copolymer systems, illustrate that variations in macromolecular architecture can yield novel NP based delivery vehicles.

## 6. Conclusions

A diverse collection of nanosized aggregates can be obtained from macromolecular architectures derived from dendron-polymer conjugate based on simple combinations of dendritic and polymeric building blocks. Since their inception more than two decades ago, advances in synthetic polymer and dendrimer chemistry have witnessed the evolution of these dendron-polymer conjugate based nanosized aggregates as simple containers for therapeutic agents to smart drug delivery systems. The abovementioned examples provide a glimpse of how clever design of the discrete dendron-polymer conjugate yields through self-assembly a ‘more than the sum’ aggregate that brings novel attributes which qualifies these nanoparticles as viable drug delivery platforms. The confluence of organic chemistry and polymer science is enabling the design of dynamic constructs that are responsive to intrinsic and external cues to yield stimuli-responsive systems. Incorporation of bioactive motifs onto these nano-objects which would allow more specificity for triggered release will further advance these systems. To date, while the efficiency of most systems have been evaluated in vitro, a few examples in recent years have demonstrated through in vivo studies the promise that these self-assembled nanosized aggregates hold for shaping the future of nanotherapeutics. One of the challenges in the area of nanoparticle technology relates to large scale synthesis of the particles with homogenous properties such as size and polydispersity. For developing practical applications, it is important that delivery vehicles can be prepared in large amounts within certain specifications for preclinical and clinical test phases. To address such issues, may be new technologies such as continuous manufacturing techniques such as use of microfluidic flow systems can provide access to large amounts of homogenous nanoparticles. For a chemist, the flexibility in the chemical design of both dendritic and polymeric components, as well as the final dendron-polymer conjugates offers plenty of room for creativity to tailor effective drug delivery platforms.
